# Influence of Participant Perceptions of Adherence-Related Interactions with Study/Study Team on Drug Levels: HPTN069 Analysis of Self-Reported Adherence Experiences While on Study

**DOI:** 10.1007/s10461-023-04215-9

**Published:** 2023-11-10

**Authors:** K. R. Amico, K. H. Mayer, R. J. Landovitz, M. Marzinke, C. Hendrix, M. McCauley, T. Wilkin, R. Gulick, HPTN 069/A5305 study team

**Affiliations:** 1 Department of Health Behavior and Health Education, School of Public Health, University of Michigan, Ann Arbor, USA; 2 Fenway Health and Harvard Medical School, Boston, USA; 3 David Geffen School of Medicine at UCLA, Los Angeles, USA; 4 Johns Hopkins University School of Medicine, Baltimore, USA; 5 FHI360, Durham, USA; 6 Weill Cornell Medicine, New York, USA

**Keywords:** PrEP, Adherence, Person-centered, Participant-centered, Drug concentration, HPTN069

## Abstract

Adherence to HIV pre-exposure prophylaxis (PrEP) study drug is critical for safety, tolerability, and efficacy trials, and may be affected by how adherence is communicated by the study staff to trial participants. Increasingly, clinical trials investigating PrEP are creating and implementing ‘participant-centered’ approaches that discuss potential non-adherence neutrally (without negative judgement) and support efforts to adhere versus insisting on perfect adherence. In the HPTN069/ACTG A5305 study, we evaluated participant experiences of potentially negative adherence-related interactions with study teams using ten items to characterize the frequency of such experiences. We related these individual items and a combined set of seven negative experience items (total negative experience score) to drug concentrations (detectable or consistent with daily-dosing). The exploratory analyses used logistic regression for each experience item on the full sample and disaggregated by sex. Several experiences were related to drug detection and to daily-dosing, although more so for participants identifying as men than women. Total negative experience scores associated with not having detection drug concentrations for the full sample, and remained significant even when controlling for sex, age, and race. Daily dosing was associated with total negative experience score for men in the sample. Additional investigations into adherence-related interactions with study teams that are most problematic or helpful in general and uniquely for men and women are warranted.

## Introduction

Implementation, delivery and options for pre-exposure prophylaxis (PrEP) for HIV prevention have progressed substantially since the FDA’s approval of oral, daily TDF/FTC for individuals at increased risk for HIV in the US in 2012 [1]. Now featured prominently in the US strategy to End the HIV/AIDS Epidemic [2], supporting individuals opting to use PrEP as part of their HIV prevention plan remains an important area of practice and study. In the context of randomized controlled trials, numerous approaches were implemented to support participant adherence [3]. These include counseling, drug concentration feedback, and real-time monitoring. Recommendations to adopt non-judgmental, neutral approaches to discussing adherence and non-adherence with study participants are now common [4], driven in part by qualitative studies that identified participant concerns about negative reactions to non-adherence as eroding engagement in study participation [3, 5]. Understanding the potential impact of interactions around adherence in the context of study participation using objectively measured adherence can help the conduct of future trials, as well as PrEP implementation, as monitoring and discussing adherence remains a strong CDC PrEP guideline recommendation [6, 7]. To date, however, even in the context of controlled trials, there have been few quantitative evaluations of how objectively measured adherence relates to perceptions of how study teams interact with participants around adherence and non-adherence.

HPTN 069/ACTG 5305: The Next PrEP Study [8, 9] investigated the feasibility, acceptability and safety of maraviroc (MVC) containing PrEP regimens among men who have sex with men and cis and transgender women. Over 48 weeks, participants in this multisite U.S. study engaged with clinical research site team members at multiple study visits, where HIV-testing, re-issuing of study drug regimen, and adherence check-ins were provided. All sites engaged in trainings focused on discussing adherence and related challenges non-judgmentally (neutral approach), which was also emphasized in study material and team meetings. Results of the study’s primary outcomes are reported elsewhere [9]. To characterize participant experiences around adherence during the study, we evaluated survey data collected at the end of study participation identifying the presence or absence of specific adherence-related experiences with study team members, overall and disaggregated by gender women, to determine if negative adherence-related experiences associated with adherence, as assessed by drug concentrations.

## Methods

HPTN069/ACTG 5305 participants completed a computer assisted survey instrument (CASI) in semi-private locations at several points throughout the 48-week Phase II double-blinded study. Participants were randomized to receive a 3-pill, daily regimen containing MVC alone, MVC + tenofovir disoproxil fumarate (TDF), MVC + emtricitabine (FTC), or FTC/TDF and/or matching placebos. The study protocol and related training asked study teams to support adherence through conversations that did not start with an assessment of missed or taken doses, that promoted reporting of difficulties with adherence by framing adherence as difficult and missed doses as not uncommon, and to avoid punishing or negative responses to reports of adherence problems.

### Measures

#### Adherence Related Experiences on Study

At the final study visit, participants were asked a set of questions on the CASI about their adherence-related experiences in the study. These 10 study-specific items listed statements about experiences or attitudes about study level support or lack of support for adherence in general and specifically in terms of participant-centered, neutral framing of adherence discussions. Response options were “yes” or “no” although participants could choose to skip any item. A subscale using 7 of these items that reflected potentially negative experience or those counter to the adherence-framing approach adopted by the study were summed into a ‘negative experiences’ scale.

#### Objectively Measured Drug Exposure/Adherence

Drug levels were evaluated on plasma samples at weeks 24 and 48. Those collected closest to final study visit were used in the current analyses. For each participant, drug detection was defined for each of their active drugs as follows: No drug detected (below limits of quantitation: BLQ) (MVC, FTC, or TFV of <  = 0.0), low levels (> 0.0 to 10.0), moderate/medium levels (> 10.0 to 40.0 for MVC and TFV, > 10.0 to 65.0 for FTC) and high (> 40 for MVC and TFV, > 65 for FTC). Participants were classified for analyses as having a drug in the detectable range or not, and as having medium/high concentration of drug or not. For participants receiving more than one active study drug, they were classified as “detectable” if any drug was detectable. Similarly, to classify those in the medium/high group, participants on more than one active study drug only needed to reach this classification of one of the drugs to qualify.

### Statistical Analyses

Item distributions were characterized to estimate how common or uncommon different adherence-related experienced were across all participants. Literature exploring disparities in health in the US suggest potential differences in interactions with care-providers for women [10, 11], younger patients [12], and Black/African American patients [13, 14]. To identify potential differences in specific experiences and interactions with study teams by sex, age group (25 or older versus younger), or race, simple logistic regression was used to characterize potential group differences. Endorsement of a given adherence-related experience item was then evaluated for association with odds of having drug detected and with odds of having drug concentrations reflecting daily dosing using simple univariate logistic regression. The parent study (HPTN069) was conducted as two cohorts- the first cohort was open to cisgender men who have sex with men and transgender women and the second enrolling cisgender women. Literature across disease areas suggests consistent differences in adherence and experiences with non-adherence more specifically between men and women, leading to recommendations to consider gender in adherence related work [15–17]. To identify potentially unique experiences between men and women in the study, we supplemented the analysis of the full sample with analyses within self-reported gender. Due to the small number of transgender individuals in our sample, we were unable to include transgender as a separate gender category. For the disaggregated analyses, we considered a number of potential strategies resulting in varying degrees of misrepresentation (e.g., combining transgender women with MSM or cisgender women) with exclusion (e.g., not including transgender participants in either group resulting in exclusion from the analyses). Opting for inclusion as the priority, we used current identification as a man or a woman (a single binary gender variable) in the disaggregated analyses. Finally, all items reflecting negative experiences were summed into a 7-item scale and evaluated for association with drug detection and concentrations reflecting daily dosing, overall and disaggregated by sex. Identified association(s) between scale score and either drug detection or concentrations suggesting daily-dosing were further evaluated to determine if negative adherence-related experiences remained significantly associated with drug concentrations while controlling for sex, age group and race. All analyses were for hypothesis generation, we did not control for inflation in error-rate.

## Results

Among the 594 HPTN069 participants, 503 had sociodemographic and support-experiences data and, of these, 393 had drug concentration data. Those missing data did not differ from those with complete data on demographics or support-experiences, with the exception of men having a higher percent missing (26%) compared to women (14%) (p = 0.005). This sample (N = 393) was 41% Black/African American (N = 160), 18% (N = 71) Latino/a, and 19% (N = 75) under the age of 25. Reported gender identity was mostly cisgender-male (64%), followed by cisgender-female (35%), transgender women (1%) and the remaining participants self-identified as other (< 1%).

### Adherence-Related Experiences with Study and Study Team – Full Sample

All (100%) participants felt well informed about how to take medications and nearly all felt informed about when to take study medications. Reported experiences that would be considered counter to the planned adherence support approach, such as feeling study team members got upset with participant non-adherence, having an unpleasant visit if reporting non-adherence, or reporting ‘no’ to feeling study team members at the site were understanding about times when doses may have been missed, were generally uncommon in the full cohort (Table 1). Negative experiences reported by over 10% of participants included feeling there was ‘pressure from the study team to take the medication’ (14%), ‘there was too much focus on needing to take the medications perfectly’ (12%), ‘there was not enough discussion about how it was going for participants with the medications’ (11%) and that there could have been more support around trying to take the study medications (11%). A large majority (83%) reported being asked about missed doses at clinical visits. Overall, most participants experienced adherence support and did *not* have negative experiences when reporting non-adherence.

**Table 1:** Distributions of reported experiences and drug detection and daily dosing

ITEM		Full sample		Drug detected				Daily dosing levels			
				No (107)		Yes (286)		No (220)		Yes (173)	
		N	%	N	%	N	%	N	%	N	%
Participants got clear information about how to take the medications	No	0	0	0	0	0	0	0	0	0	0
	Yes	392	100	106	100	286	100	219	100	173	100
	Total	392	100	106	100	286	100	219	100	173	100
Participants got clear information about when to take the medications	No	8	2.0	4	4	4	1	4	2	4	2
	Yes	385	98	103	96	282	99	216	98	169	98
	Total	393	100	107	100	286	100	220	100	209	100
*Participants felt pressured from the study team to take the medications*	No	334	86	88	84	246	86	182	84	152	88
	Yes	56	14	17	16	39	14	35	16	21	12
	Total	390	100	105	100	285	100	217	100	173	100
*People got upset with participants when medications were not taken as recommended/prescribed*	No	359	92	**92**	**86**	**267**	**95**	**195**	**90**	**164**	**96**
	Yes	29	8	**15**	**14**	**14**	**5**	**22**	**10**	**7**	**4**
	Total	388	100	**107**	**100**	**281**	**100**	**217**	**100**	**171**	**100**
Study team members did not ask about missed doses of study medication	No	320	83	88	83	232	83	177	82	143	84
	Yes	66	17	18	17	48	17	38	18	28	16
	Total	386	100	106	100	280	100	215	100	171	100
*If participants reported missing a dose of study medications, the study visit became unpleasant*	No	364	94	97	92	267	95	202	94	162	95
	Yes	22	6	8	8	14	5	13	6	9	5
	Total	386	100	105	100	281	100	215	100	171	100
*Staff were understanding about times when people didn’t take the study pills*	No	31	8	**14**	**13**	**17**	**6**	**25**	**12**	**6**	**4**
	Yes	354	92	**91**	**87**	**263**	**94**	**191**	**88**	**163**	**96**
	Total	385	100	**105**	**100**	**280**	**100**	**216**	**100**	**169**	**100**
*There was too much focus on participants needing to take the medications perfectly*	No	337	88	**85**	**83**	**252**	**90**	186	88	151	88
	Yes	46	12	**18**	**17**	**28**	**10**	26	12	20	12
	Total	383	100	**103**	**100**	**280**	**100**	212	100	171	100
*There was not enough discussion about how it was going for participants with the medications*	No	346	89	89	86	257	90	191	89	155	90
	Yes	42	11	15	14	27	10	24	11	18	10
	Total	388	100	104	100	284	100	215	100	173	100
*The study team could have been more supportive when it came to participants trying to take the medications*	No	342	89	**82**	**80**	**260**	**93**	188	86	154	91
	Yes	41	11	**21**	**20**	**20**	**7**	26	14	15	9
	Total	383	100	**103**	**100**	**280**	**100**	214	100	169	100.0

*Negative or counter neutral-approach experiences in italics*

Significant difference between those reporting and those not reporting the experience on drug concentration outcome bolded

### Demographic Differences in Adherence-Related Experiences with Study and Study Team

Gender, age group and race were evaluated for association with odds of endorsing each of the adherence-experience items. Cisgender women (n = 138, 35% of sample) in comparison to other identifying participants (n = 253) were generally comparable in endorsement of survey items about experiences with the study staff in relation to adherence counseling except for two items. Cisgender Women were significantly more likely to report (compared to cisgender men) that study team members did not ask about missed doses of study medication (OR 1.98 (1.15, 3.39), p = 0.01, 23% versus 13%) and that there was too much focus on participants needing to take the medications perfectly (OR 2.99 (1.59, 5.61), p = 0.001, 20% versus 8%). For participants less than 25 years of age (n = 75, 19% of the sample), the only significant differences were for higher endorsement of ‘people got upset with participants when medications were not taken as recommended’ (OR 2.38 (1.06, 5.36), p = 0.04, 13% versus 6%) and ‘the study team could have been more supportive when it came to participants trying to take the medication’ (OR 2.10 (1.03, 4.28), p = 0.04, 17% versus 9%). White participants (n = 202, 51% of sample) generally did not significantly differ from other races (n = 191) in endorsement of any item. African American/Black participants (n = 160, 41% of sample) were similar in comparison to other races (n = 233) in item endorsement with the exception of slightly higher endorsement of feeling that ‘people got upset with participants when medications were not taken as recommended’ (OR 2.16 (1.00, 4.67), p = 0.05, 11% versus 5%).

### Drug Detection and Daily Dosing Concentrations

By regimen, FTC concentrations were evaluated for 210 participants, which identified 27% with undetectable drug (no dosing), 27% at levels that were detectable but less than daily, and 46% at levels suggesting daily dosing. For TFV concentrations among 205 participants, 29% were not detectable (no evidence of dosing in the past 48–72 h), 20% were detectable but less than daily, and 51% were daily. For MCV among 286 participants, 35% were undetectable, 40% detectable but less than daily, and 25% had concentrations suggesting daily dosing. Overall, 27% of participants (n = 107) were classified undetectable, 29% of participants (n = 113) had drug detected but at less than daily levels, and 44% of participants (n = 173) had at least one drug concentration at the daily-dosing cut-off.

### Drug Detection and Daily Dosing Concentration by Reported Adherence-Related Experiences with Study and Study Team

Item response by detection of drug and by concentrations suggesting daily dosing are presented in the Table 1 for the full sample. Drug detection was associated with lower reports of feeling people got upset when medications were not taken (OR 0.32 (0.15, 0.69), p < 0.01), feeling there was too much focus on participants needing to take the medications perfectly (OR 0.53 (0.28, 0.99), p = 0.05), and feeling the study team could have been more supportive when it came to participants trying to take the medication(s) (OR 0.30 (0.16, 0.58), p < 0.001), and a higher percent of participants reporting that staff were understanding about missed doses (OR 2.38 (1.13, 5.02), p = 0.02). Responses associated with having drug concentration consistent with daily dosing included lower reporting of feeling people got upset when non-adherence was reported (OR 0.38 (0.16, 0.91), p = 0.03) and higher reporting that staff were understanding about missed doses (OR 3.56 (1.42, 8.88), p < 0.01).

### Experiences and Drug Concentration Level Disaggregated for Men and Women

Table 2 disaggregates associations between experiences at site by self-reported gender; 253 men (252 cisgender men and 1 transgender man) and 138 women (136 cisgender and 2 transgender women). Two participants identifying as “other” were not included in these analyses. For any drug detected, associations between reported experiences with study medication adherence support were only present among men. In that group, perceptions that people got upset with participants when medications were not taken distinguished between those with no drug detected (21% endorsing this belief) and those who had drug detected (with only 3% of those with detectable drug endorsing the belief), OR 0.11 [0.04, 0.34], p < 0.001. Similarly, endorsing experiences regarding visits becoming unpleasant if missing a dose was reported (OR 0.23 [0.07, 0.79], p = 0.02), too much focus on participants needing to take medications perfectly (OR 0.31 [0.12, 0.85], p = 0.02), and feeling the study team could have been more supportive in terms of participants trying to take medications was higher in the group without drug detection (OR 0.12 [0.05, 0.30], p < 0.001). More men in the drug detectable group reported they felt the staff were understanding about times when people did not take study pills (94%) than those who did not have drug detected (87%), OR 3.41 [1.23, 9.34], p = 0.02. No associations were identified for experiences and drug detection for women. For daily dosing in the male group, feeling people got upset when medications were not taken was the only item demonstrating a significant association (OR,13 [0.03, 0.59], p < 0.01). For women, feeling there was too much focus on needing to take study medications perfectly was associated with greater odds of being in the daily-dosing group, with 15% in the less than daily dosing group endorsing this belief and 32% of those in the daily-dosing group reporting it (OR 2.65 [1.11, 6.33], p = 0.03).

**Table 2:** Experiences and Drug Concentration for Women and Men (negative or counter neutral-approach experiences in italics)

ITEM		Men (253)								Women (138)							
		Drug detected				Daily dosing levels				Drug detected				Daily dosing levels			
		No (43)		Yes (210)		No (120)		Yes (129)		No (63)		Yes (75)		No (95)		Yes (41)	
		N	%	N	%	N	%	N	%	N	%	N	%	N	%	N	%
Participants got clear information about how to take the medications	No	0		0		0				0		0					
	Yes	42	100	210	100	121	100.0	131	100.0	63	100	75	100	97	100.0	41	100.0
	Tot	42	100	210	100	121	100.0	131	100.0	63	100	75	100	97	100.0	41	100.0
Participants got clear information about when to take the medications	No	2	4.7	4	1.9	2	1.6	4	3.1	2	3.2	0	0.0	2	2.1	0	0.0
	Yes	41	95.3	206	98.1	120	98.4	127	96.9	61	96.8	75	100.0	95	97.9	41	100.0
	Tot	43	100.0	210	100.0	122	100.0	131	100.0	63	100.0	75	100.0	97	100.0	41	100.0
*Participants felt pressured from the study team to take the medications*	No	33	78.6	184	87.6	100	82.6	117	89.3	54	87.1	61	82.4	81	85.3	34	82.9
	Yes	9	21.4	26	12.4	21	17.4	14	10.7	8	12.9	13	17.6	14	14.7	7	17.1
	Tot	42	100.0	210	100.0	121	100.0	131	100.0	62	100.0	74	100.0	95	100.0	41	100.0
*People got upset with participants when medications were not taken as recommended/prescribed*	No	**34**	**79.1**	**200**	**97.1**	**107**	**89.2**	**127**	**98.4**	57	90.5	66	89.2	87	90.6	36	87.8
	Yes	**9**	**20.9**	**6**	**2.9**	**13**	**10.8**	**2**	**1.6**	6	9.5	8	10.8	9	9.4	5	12.2
	Tot	**43**	**100.0**	**206**	**100.0**	**120**	**100.0**	**129**	**100.0**	63	100.0	74	100.0	96	100.0	41	100.0
Study team members did not ask about missed doses of study medication	No	36	83.7	178	87.3	100	84.7	114	88.4	51	82.3	54	72.0	76	79.2	29	70.7
	Yes	7	16.3	26	12.7	18	15.3	15	11.6	11	17.7	21	28.0	20	20.8	12	29.3
	Tot	43	100.0	204	100.0	118	100.0	129	100.0	62	100.0	75	100.0	96	100.0	41	100.0
*If participants reported missing a dose of study medications, the study visit became unpleasant*	No	**38**	**88.4**	**199**	**97.1**	111	93.3	126	97.7	58	95.1	67	89.3	90	94.7	35	85.4
	Yes	**5**	**11.6**	**6**	**2.9**	8	6.7	3	2.3	3	4.9	8	10.7	5	5.3	6	14.6
	Tot	**43**	**100.0**	**205**	**100.0**	119	100.0	129	100.0	61	100.0	75	100.0	95	100.0	41	100.0
*Staff were understanding about times when people didn’t take the study pills*^*a*^	No	**7**	**16.3**	**11**	**5.4**	**14**	**11.7**	**4**	**3.1**	7	11.5	6	8.0	11	11.6	2	4.9
	Yes	**36**	**83.7**	**193**	**94.6**	**106**	**88.3**	**123**	**96.9**	54	88.5	69	92.0	84	88.4	39	95.1
	Tot	**43**	**100.0**	**204**	**100.0**	**120**	**100.0**	**127**	**100.0**	61	100.0	75	100.0	95	100.0	41	100.0
*There was too much focus on participants needing to take the medications perfectly*	No	**35**	**83.3**	**192**	**94.1**	105	89.7	122	94.6	49	81.7	59	78.7	**80**	**85.1**	**28**	**68.3**
	Yes	**7**	**16.7**	**12**	**5.9**	12	10.3	7	5.4	11	18.3	16	21.3	**14**	**14.9**	**13**	**31.7**
	Tot	**42**	**100.0**	**204**	**100.0**	117	100.0	129	100.0	60	100.0	75	100.0	**94**	**100.0**	**41**	**100.0**
*There was not enough discussion about how it was going for participants with the medications*	No	36	83.7	188	90.4	106	88.3	118	90.1	52	86.7	68	90.7	84	89.4	36	87.8
	Yes	7	16.3	20	9.6	14	11.7	13	9.9	8	13.3	7	9.3	10	10.6	5	12.2
	Tot	43	100.0	208	100.0	120	100.0	131	100.0	60	100.0	75	100.0	94	100.0	41	100.0
*The study team could have been more supportive when it came to participants trying to take the medications*	No	**31**	**72.1**	**197**	**95.6**	106	88.3	122	94.6	50	84.7	63	86.3	81	87.1	32	82.1
	Yes	**12**	**27.9**	**9**	**4.4**	14	11.7	7	5.4	9	15.3	10	13.7	12	12.9	7	17.9
	Tot	**43**	**100.0**	**206**	**100.0**	120	100.0	129	100.0	59	100.0	73	100.0	93	100.0	39	100.0

Current gender self-reported as man (252 cis-gender and 1 transgender man) or woman (136 cis-gender and 2 transgender women)

^a^Reverse scored for negative experience

Negative or counter neutral-approach experiences in italics

Significant difference between those reporting and those not reporting the experience on drug concentration outcome bolded

### Experiences as a Scale

The summed set of 7 items reflecting potentially negative experiences summed, as available, ranged from 0 to 6. As a scale, the items were intercorrelated (Cronbach’s alpha = 0.77). Most participants (67%) reported zero experiences, 19% (n = 96) endorsed 1 item, 7% (n = 33) endorsed 2 items, and 7% (n = 37) endorsed 3 or more. Average number endorsed (0.67, SD 1.31) was lower for men (0.58, SD 1.28) than for women (0.86, SD 1.55), t(249.61) = -2.03, p < 0.05 (equal variances not assumed). In logistic regression models for all participants (Table 3), there was a significant association between total experiences and drug detection but not for daily dosing concentrations. The higher one’s negative experiences, the less likely they were to have detectable drug. Within the cisgender male group (Table 3), both drug concentration measures significantly associated with total negative experiences scores. Within the cisgender female group, there was no significant association between total experiences and drug detection or drug at daily dosing concentrations. In multivariable logistic regression models controlling for sex (male/female), age group, and African American/Black race, total negative experiences scale score remained significantly associated with detectable drug concentration (OR 0.83 [0.70, 0.99], p = 0.04).

**Table 3:** Total negative experiences score

Participants	Drug concentration group	OR	Confidence interval		P value
			LL	UL	
All participants	Drug Detectable	0.79	0.67	0.92	< .01
Men	Drug Detectable	0.60	0.46	0.77	< .001
Women	Drug Detectable	1.03	0.83	1.28	0.76
All participants	Daily Dosing	0.87	0.74	1.03	0.10
Men	Daily Dosing	0.70	0.54	0.91	< .01
Women	Daily Dosing	1.13	0.91	1.41	0.27

## Discussion

Promoting study experiences that support adherence in clinical trial participants remains a high priority. Given the accumulated qualitative literature suggesting that adherence-related communications between study team and participants can be negatively experienced and may deter adherence, understanding which experiences may impact adherence can provide guidance for operational and implementation planning. Results of our evaluation of 10 adherence-related experience items collected at the end of the HPTN069/ACTG 5305 (NextPrEP) study suggest that some experiences do relate to taking drug at all (drug detection) and to daily dosing (concentrations consistent with daily dosing). During study start-up, site research team members attended a training on overall approach to adherence, which specifically covered “neutral” and non-judgmental discussions. Across the full sample, experiences counter to the protocol-recommended approach for supporting adherence were generally uncommon. We did not observe a pattern of differences in reporting specific experiences on the basis of the age or race group variables. Feeling “people got upset with participants” in response to non-adherence was endorsed uniquely higher among young participants (under 25 years of age) and Black/African American participants. Young participants also endorsed the item about feeling staff could have been ‘more supportive’ around non-adherence more than older participants. Other items were similarly endorsed across groups.

Results specific to experiences on study and drug detection suggested several potential associations. Feeling “people got upset with participants” in response to non-adherence was reported more commonly among those with no drug detected and those not reaching daily-dosing concentration levels. Feeling that “staff were understanding about times when people didn’t take the study pills” was more common among those with drug detection and those with drug concentrations reaching daily-dosing levels. Reporting “there was too much focus on participants needing to take the medications perfectly” and that the “study team could have been more supportive” in terms of participant efforts to take study the study medications was related to not having drug detected. Results suggest that aspects of person-centeredness (e.g., focus on efforts/experiences versus missed doses) and neutrality (eg., avoiding conveying being upset or disappointed with participant non-adherence) did associate with engaging with a regimen (drug detection) and adherence (daily drug concentrations). Other experiences, like feeling pressured to take the study drug, asking about missed doses specifically, and ensuring that discussions included overall experiences with study drug (versus executed adherence) were not associated with adherence outcomes, nor were reports of feeling the study visit became “unpleasant” if missed doses were reported.

When disaggregated by gender, the cohort of men, who made up the majority of the full sample, had greater reports of negative adherence-related experiences and a greater number of these items that associated with drug detection and daily-dosing. The only scale item that distinguished those with daily-dosing among women was feeling there was too much focus on participants needing to take the medications perfectly. Women were also more likely to report this experience in comparison to men. Counter to hypotheses and opposite of results for men, more women dosing daily reported this experience compared to those with concentrations suggesting less than daily dosing, suggesting it could be a facilitator of dosing. It is unclear if the items we used to capture potentially negative experiences were interpreted in the same way between groups of men and women, or if adherence-related experiences among women were simply less associate with adherence. Work on engagement of young women in PrEP studies and programs in other regions of the world have offered valuable insights into how to best engage women [18]. Of note, our approach of remaining “neutral” around adherence aims to eliminate shaming or critique of non-adherence but also eliminates praise for high adherence. More overt and explicit reinforcement of adherence may be a source of support for women. The differences identified, in terms of higher numbers of men with drug detected and high drug concentrations and potential for unique factors influencing adherence, are consistent with literature in other areas [17], but do not provide much needed insights into how genders may differ in their experiences. Additional work in this area is warranted.

In terms of total experiences, the association between drug detection and total number of negative adherence-related experiences remained significant even when controlling for a host of demographic variables, including gender (man/woman), race, and age-group. Thus, results suggest that attending to and optimizing adherence-related interactions with study site teams may have an impact on engagement with PrEP drugs.

Overall and disaggregated by gender, our results demonstrating an association between certain experiences and drug detection and drug levels suggesting daily dosing provide direction for future investigation. Associations could reflect the impact of certain experiences on one’s engagement with the study drug, or one’s engagement or challenges with engagement with the study drug may have led to expectations for negative interactions. Over time, the relationship is likely even more complex. Our cross-sectional evaluation is not positioned to speak to directionality. Rather, the results draw attention to factors that warrant additional focused research and exploration.

To date, qualitative explorations of experiences in study trials where adherence was problematic have identified interpersonal interactions as influential on adherence, but largely in terms of personal and in-community relationships. Participants in FEM-PrEP reflected on pressure from significant others but experiences with study team members was largely characterized as very positive [19, 20]. In the iPrEX and VOICE trials, negative experiences around adherence, and potential non-adherence more specifically, led to extensive protocol revisions to adopt more person-facing, participant-centered, neutral approaches to discussing adherence and non-adherence with participants [3, 21]. In our qualitative work with women in the current study, we found women generally felt well cared for by study team members [22], which is consistent with our identification of low numbers of women reporting negative experiences around adherence and few associations between these and drug concentrations. Qualitative investigation into if and how men and women engaged in biomedical prevention studies experience adherence-related discussions with study team members could provide nuanced insights into the kinds of interactions that are helpful, that should be avoided, or that have no consistent impact on adherence.

Our study is unique in mapping experiences onto drug concentrations. However, it is limited by a sole focus on drug concentration outcomes. Multiple contextual and more proximal factors likely intersect relationships between adherence-related experiences at study site and drug concentration. For example, trust in the clinician(s) prescribing PrEP [5, 23], and in the study more generally [24], are important contextual factors that may influence how interactions at site are experienced. Community, personal, and structural factors can influence how participants interact with study teams and with the study drug(s). One’s experiences with use and non-use of study drugs may have heightened experiences with positive and negative interactions. For example, non-use may prime a participant for experiencing exploration and check-ins on ‘how things are going with study drug’ as intrusive or accusative in nature. Our exploration cannot speak to directionality or causality of observed associations. Rather, the work is intended to identify important areas for additional inquiry, which should include efforts to distill directionality and unpack the host of potential drivers, modifiers and confounders in the relationship between study staff interactions around adherence and participant experiences with adherence.

Our approach was exploratory and, as such, did not control for inflation of type-1 error rate. As such, significance of findings should be interpreted with caution and require replication in studies where such outcomes are pre-specified and appropriately powered. Our decision to disaggregate by gender defined as a binary does not provide good representation of the gender continuum. A small number of transgender women participated in HPTN069, making the transgender cohort too small in size to meaningfully characterize. Results from the full sample should be generalized to transgender women with caution. Results from the disaggregated presentation for cisgender men who have sex with men are likely most robust and generalizable, given the sample size.

It is important to draw attention to the items we used to characterize experiences. These items were created for the current study and captured participant reflections on experiences which will include beliefs, anticipated reactions, and related implicit and explicit bias only at the final study visit. Some items, like being asked about missed doses, present interpretation challenges. For example, one item asked participants to report on *not* being asked about missed doses. Participants may have struggled with this wording. Even with accurate understanding of the item, being asked about missed doses may have occurred in otherwise supportive conversations. Development of items offering better interpretability is warranted in future investigations. Expanding the assessment approach to include objective measures could also complement self-report strategies. We did not objectively measure the occurrence or lack of occurrence of messages or types of communications between clinic staff and participants. Thus, adherence-related experiences in our work are more appropriately understood as *perceptions* of adherence-related experiences. Moreover, these would likely need to be highly saliant experiences because participants reported on these at the final assessment study visit, representing nearly a year of interactions with study team members. To minimize recall bias and memory demands, future work should incorporate periodic assessments over time.

Our data reflect experiences with study drug, versus open-label PrEP, in the context of a large, multisite clinical trial, and may be limited in application to real-world PrEP programs or demonstration projects. Research studies create unique settings. However, given the critical impact of study-drug adherence on the outcomes, conclusions and utility of research, focused investigations into the potential impact of study features (e.g., procedures, interactions with study team members) on participant behaviors (e.g., study product uptake and adherence) are needed.

## Conclusion

The set of 10 adherence-experience items provided valuable information about participant perceptions of how adherence was framed, non-adherence was reacted to, and participants were supported in the HPTN069 NextPrEP study. Given that some experiences associated with drug concentration outcomes, attention to site-level interactions around adherence in clinical trial protocols and standards of procedures appears warranted. Drug exposure is critical in safety, tolerability and efficacy trials and PrEP adherence correlates with prevention efficacy of PrEP products. Despite this, non-adherence will be an issue for some, as it is with any other prevention or treatment intervention [25]. Clinical trial planning should include community-engaged efforts to identify the content and tone of study-specific communications, messaging and interactions around adherence that have high acceptability. Identifying best practices for framing, discussing, and working with non-adherence, in general and within specific key populations, should be prioritized in ongoing and future PrEP clinical trials and projects.
